# Phenotypic and genotypic changes of *Staphylococcus aureus* in the presence of the inappropriate concentration of chlorhexidine gluconate

**DOI:** 10.1186/s12866-022-02522-0

**Published:** 2022-04-13

**Authors:** Neda Baseri, Shahin Najar-Peerayeh, Bita Bakhshi, Floriana Campanile

**Affiliations:** 1 Department of Bacteriology, Faculty of Medical Sciences, Tarbiat Modares University, Tehran, Iran; 2 Department of Biomedical and Biotechnological Sciences (BIOMETEC), Medical Molecular Microbiology and Antibiotic Resistance Laboratory (MMARLab), University of Catania, Catania, Italy

**Keywords:** Vancomycin-Intermediate Staphylococcus aureus, Regulatory genes, Disinfectant, Biguanide, Antimicrobial drug resistance

## Abstract

**Background:**

Chlorhexidine gluconate (CHG) is a disinfectant agent with different applications in health care. Improper use of CHG causes antimicrobial resistance in bacteria as a public health threat. Since *Staphylococcus aureus* is a common bacteria, it is expected usually exposed to CHG in the hospital and community. The present study aimed to correlate the phenotypic and genotypic changes in a *S. aureus* strain upon serial adaptation with supra-inhibitory CHG concentration for 50 days.

**Results:**

After in vitro serial culture of 5 × 10^5^ CFU/ml of a clinical vancomycin-susceptible *S. aureus* strain (VAN-S) into brain heart infusion (BHI) broth containing CHG 1/4, 1/2, 1, and 2 × minimal inhibitory concentration (MIC) values of VAN-S in 37 °C during 50 days, we isolated a *S. aureus* strain (CHG_Van-I_) with a ≥ twofold decrease in susceptibility to CHG and vancomycin. CHG-induced CHG_Van-I_ strain was considered as a vancomycin-intermediate *S. aureus* (VISA) strain with a VAN MIC of 4 μg/ml using the broth macro dilution method. However, reduced resistance was observed to tetracycline family antibiotics (doxycycline and tetracycline) using a modified Kirby-Bauer disk diffusion test. Moreover, a remarkable reduction was detected in growth rate, hemolysis activity (the lysis of human red blood cells by alpha-hemolysin), and colony pigmentation (on BHI agar plates). Biofilm formation (using the Microtiter plate method and crystal violet staining) was significantly increased upon CHG treatment. Adaptive changes in the expression of a set of common genes related to the development of VISA phenotype (*gra*TSR, *vra*TSR, *wal*KR, *agr* RNAIII, *sce*D, *pbp*B, and *fmt*A) were analyzed by Reverse Transcription quantitative PCR (RT-qPCR) experiment. Significant changes in *vra*TSR, *agr* RNAIII, *sce*D, and *pbp*B expression were observed. However, gene sequencing of the two-component system *vra*TSR using the Sanger sequencing method did not detect any non-synonymous substitution in CHG_Van-I_ compared to wild-type. The clonality of VAN-S and CHG_Van-I_ strains was verified using the pulsed-field gel electrophoresis (PFGE) method.

**Conclusions:**

The importance of the present study should be stated in new detected mechanisms underlying VISA development. We found a link between the improper CHX use and the development of phenotypic and genotypic features, typical of VISA clinical isolates, in a CHG-induced strain.

Since disruption of the cell wall biosynthesis occurs in VISA isolates, our CHG-induced VISA strain proved new insights into the role of CHG in the stimulation of the *S. aureus* cell wall.

## Background

Chlorhexidine gluconate (CHG) is used as a disinfectant, preservative, and constructive component in cosmetic products. There have been reports of its inefficiency in infections control which can be due to misuse of CHG concentrations [1]. It has been predicted that CHG may lead to the development of new clones with reduced susceptibility to antimicrobial agents and increased biofilm formation in bacteria, including in *Staphylococcus aureus* [2].

Recently, we emphasized that exposure to a sub-inhibitory concentration of CHG induced the development of vancomycin-intermediate *S. aureus* (VISA) with the common phenotypic and genotypic features of clinical VISA isolates, including increased biofilm formation, and decreased autolytic and hemolytic activities. Furthermore, we suggested that CHG may affect walKR and vraTSR regulatory systems [3].

The increasing prevalence of VISA strains is one of the major public health problems. VISA can cause life-treating infections such as hospital-acquired bloodstream infections [4]. In VISA strains, attenuated virulence such as suppression of hemolysis, and increased biofilm formation is a stealth strategy for surveillance, evade host immunity, and promote chronic infection. These changes are related to genetic alteration in isolates [5, 6]. Commonly, observed genetic and transcriptomic changes in VISA strains occurred in regulatory genes such as *wal*KR, *vra*TSR, and *gra*SR systems [7].

The present study aimed to support the previous observations that inappropriate inhibitory concentrations of CHG can lead to reduced susceptibility to vancomycin (VAN) with genetic changes. In the previous study, *S. aureus* was exposed to the sub-inhibitory minimal inhibitory concentration (MIC) value of CHG [3]. Here, we exposed another *S. aureus* clinical strain to the supra-inhibitory concentrations of CHG. Then, phenotypic and genotypic changes were examined. The importance of the cause of VISA development and the effect of CHG on the cell wall is not considered (except for our previous study). Therefore, more studies are needed to show the importance of this topic.

## Methods

### Bacterial strain and inductive chemical substance

To investigate induced phenotypic and genotypic changes by exposure to CHG in *S. aureus*, we used a VAN-sensitive MRSA strain, called the wild-type VAN-S strain. VAN-S strain was isolated from a burn wound of a hospitalized patient in Motahhari Hospital, Tehran, Iran, in December 2018. This strain had a CHG MIC value of 0.0625 μg/ml.

Recently, we reported a VISA strain derived from the parental strain used in the present study (VAN-S) by in vitro exposure to VAN [8].

Chlorhexidine gluconate 20% wt/vol (Sigma, Germany) was used for the present experiment. A stock solution of CHG was prepared at a concentration of 16 μg/ml, and dilutions (in sterile distilled water) were made as to the desired concentrations (see next section).

### In vitro serial exposure of *S. aureus* to CHG

Wild-type *S. aureus* VAN-S was exposed to supra-inhibitory concentrations of CHG according to a previously described adaptation protocol with minor modification [8]. Briefly, overnight culture of wild-type strain was adjusted to a starting inoculum density of 5 × 10^5^ CFU/ml in BHI (brain heart infusion) broth, exposed to CHG 1/4 × MIC value for VAN-S (0.015 μg/ml), and incubated at 37 °C for 24 h. Then, the cultures were inoculated (5 × 10^5^ CFU/ml) into BHI broth containing CHG 1/2 × MIC value (0.031 μg/ml) with described above incubation conditions. For the next rounds of passaging on days 3 and 4, the cultures were exposed to CHG 1 and 2 × MIC values (0.0625 μg/ml and 0.125 μg/ml), respectively. The adaptation process was repeated from step 1 to end within 50 days. Cultures from the 50th day were passaged on BHI agar plates without CHG over five passages and then preserved as mutant strain CHG_Van-I_ at -20 for subsequent analysis.

For quality control, VAN-S strain was also passaged into a media without CHG for 50 days in parallel culturing into the media containing CHG.

### Antimicrobial susceptibility testing

Antimicrobial susceptibility tests were performed in cation-adjusted Mueller–Hinton medium (Merck, Germany), according to the 2018 CLSI instructions [9]. *S. aureus* ATCC® 25,923 was used as a control strain. After serial CHG exposure, the changes in antibacterial susceptibility were interpreted as the fold change (≥ twofold) relative to the wild-type VAN-S strain.

The CHG and VAN MIC values of wild-type VAN-S and mutant CHG_Van-I_ strains were monitored using the broth macro dilution method.

Susceptibility to cefoxitin (30ug), chloramphenicol (30 μg), ciprofloxacin (5 μg), clindamycin (2 μg), doxycycline (30 μg), erythromycin (15 μg), gentamicin (10 μg), linezolid (30 μg), mupirocin (5 μg), ofloxacin (5 μg), rifampicin (5 μg), tetracycline (30 μg), and trimethoprim-sulfamethoxazole (1.25 μg / 23.75 μg) were determined using modified Kirby-Bauer disk diffusion test.

### Verification of clonality between wild-type and CHG-induced mutant strains

After the selection of CHG-induced CHG_Van-I_ strain, the clonality of wild-type VAN-S and mutant CHG_Van-I_ strains was verified using pulsed-field gel electrophoresis (PFGE) on a CHEF-Mapper (Bio-Rad, USA) system as described previously [10]. *Salmonella* ser. Braenderup H9812 was used as a standard strain. *Sma*I and *Xba*I restriction endonucleases (Takara, Japan) were used for digesting the tested and standard strains, respectively.

### Quantification expression of candidate genes involved in VISA mechanism

Gene expression of the most common loci and genes known to be involved in the VISA mechanism, including *gra*TSR, *vra*TSR, *wal*KR, *agr* RNAIII, *sce*D, *pbp*B, and, *fmt*A were quantified in wild-type VAN-S and mutant CHG_Van-I_ strains using the Reverse Transcription quantitative PCR (RT-qPCR) method. The *gyr*A gene was used as an internal control gene.

For this purpose, RNA was extracted (GeneAll Hybrid-R™ RNA isolation kit, Korea) from mid-logarithmic phase cultures, then treated with DNase I (Thermo Fisher Scientific, USA) and reversed to cDNA (Yekta Tajhiz Azma cDNA synthesis kit, Iran). The RT-qPCR was carried out in a Rotor-Gene Q (Qiagen, Germany) with the cDNA template in the final concentration of 5 ng, SYBR Green (Ampliqon, Denmark), and previously designed primers (Pishgam Biotech, Iran) [3, 11–14].

The RT-qPCR experiment was independently performed three times. Relative expression data were analyzed using the Relative Expression Software Tool (REST) 2009 (v2.0.13; Qiagen, USA) by the ∆∆Ct method. The pairwise fixed randomization test with 2000 iterations (as randomization number) in a rest standard mode was used.

### Complete sequencing of candidate transcriptional regulatory genes

WalKR, VraTSR, and GraSR are the main transcriptional regulator systems in *S. aureus*. The *vra*S and *vra*T genes were selected for further sequence analysis due to modification in their gene expression levels (see the results of the RT-qPCR analysis).

Genomic DNA extraction was performed according to the Gene Transfer kit (Pioneers, Iran) instructions. The complete sequence of *vra*S and *vra*T genes were amplified by PCR in a T100™ Thermal cycler (BioRad, USA) using Taq DNA Polymerase 2 × Master Mix (Ampliqon, Denmark) and previously published primers [3].

After verifying using gel electrophoresis, the PCR products were purified and sequenced by Microsynth AG company (Switzerland).

The sequence read of CHG-induced CHG_Van-I_ strain was compared to wild-type VAN-S strain using the alignment in allele ID software (V6.00, USA) and BLAST database (https://blast.ncbi.nlm.nih.gov/Blast.cgi) to find CHG-induced single nucleotide polymorphisms (SNPs).

### Growth and autolysis kinetics analysis

Growth and autolysis kinetics were analyzed according to the previously described protocols [15, 16] summarized here. For growth kinetic analysis, cultures were adjusted to an optical density at 660 nm (OD_660_) of 0.01 in BHI broth and incubated at 37 °C for 15 h. To obtain the logarithmic phase values at the start and final time points, the changes in OD_660_ were recorded using a spectrophotometer (Biochrom WPA Biowave II, UK) at one-hour intervals. The doubling time was calculated using the previously defined formula [15].

For autolysis kinetic, washed bacteria cells were added to cold distilled water supplemented with 0.1% Triton X-100 to reach an OD_600_ of 1. The cultures were incubated at 37 °C with shaking at 200 rpm for 4 h, and the OD_600_ values were read at one-hour intervals by spectrophotometer [16].

The experiments were performed at three independent times. All data were statistically analyzed using SPSS24 statistical software (SPSS inc., Chicago, IL). A *P* value ≤ 0.05 was considered as a significant difference.

The growth kinetic data were compared between wild-type VAN-S and mutant CHG_Van-I_ strains using the nonparametric two-tailed Wilcoxon signed-rank test. Parametric paired-sample Student’s *t*-test was used to calculate autolysis activity.

### Biofilm formation analysis

The in vitro evolution of the biofilm formation was conducted using the microtiter plate method as previously described [17]. Briefly, dilated cultures in BHI broth supplemented with 0.5% (w/v) glucose with a final concentration of 10^7^ CFU/ml were distributed into wells of a 96-well polystyrene microtiter plate. After incubation at 37 °C for 24 h with shaking at 120 rpm, loosely bound cells to wells were washed with phosphate-buffered saline (PBS) solution. Crystal violet 0.1% and ethanol-acetone (80:20, wt/wt) solutions were used for biofilm staining and dye solubilizing, respectively. The microplate reader (Epson LQ-300, p852A, Japan) measured the absorbance of the final solution at OD_570_.

*S. aureus* ATCC® 25,923 and media without bacteria were used for quality control as positive and negative controls, respectively. The trials were performed in three replicates on three independent occasions and analyzed using the paired-sample Student’s *t*-test.

### Alpha-hemolysis analysis

The lysis of human red blood cell (RBC) by alpha-hemolysin of *S. aureus* was compared between wild-type VAN-S and mutant CHG_Van-I_ strains, as conducted previously with the following modifications [18].

The whole blood was obtained from a healthy volunteer. Then, RBCs were separated from plasma using centrifugation (900 × g for 2 min). The washed and diluted RBCs in PBS solution (300 μl of RBCs in 10 ml PBS) were added (50:1) to the adjusted *S. aureus* culture to an OD_600_ of 0.3 in TSB.

The mixture was incubated at 35 ± 2 °C with shaking at 250 rpm for one hour and centrifuged at 16,000 × g for 10 min. The OD_543_ value of the supernatant was measured, and then the percent of hemolysis activity was calculated using negative (diluted RBCs in PBS solution) and positive (Triton X-100) controls. Values were obtained from three independent trials with a two-tailed Wilcoxon signed-rank test analysis.

## Results

### CHG Induced changes in antibiotic susceptibility pattern of *S. aureus*

Before serial CHG exposure, the CHG and VAN MIC values for wild-type VAN-S were 0.0625 μg/ml and 1 μg/ml, respectively. After 50 days of CHG treatment, one CHG-induced mutant (CHG_Van-I_ strain) was selected with enhanced (≥ twofold) CHG and VAN MIC values. Despite the increased CHG MIC value (0.25 μg/ml), the CHG_Van-I_ strain was still in the sensitive range of CHG. However, it was considered a VISA strain with a VAN MIC of 4 μg/ml. No changes were observed in the VAN and CHG MIC values in parallel culturing into the media non containing CHG.

The results of the disk diffusion test are shown in Table 1. Interestingly, resistance to doxycycline and tetracycline was reduced (≥ twofold) after CHG treatment. No significant changes were observed in the susceptibility pattern to other antibiotics.

**Table 1:** Antibiotic susceptibility pattern of wild-type VAN-S and mutant CHG_Van-I_ strains. The inhibition growth zones of strains against antibiotics are reported as diameter

Strains	Inhibition growth zone diameter of strains against antibiotics (mm)												
	FOX	CHL	CIP	CLI	DOX	ERY	GEM	LZD	MUP	OFX	RIF	TET	SXT
**VAN-S**	16 R	22 S	25 S	22 S	0 R	0 R	20 S	25 S	22 A	22 S	26 S	0 R	26 S
**CHG**_**Van-I**_	12 R	22 S	22 S	23 S	20 S	0 R	15 S	25 S	25 A	24 S	25 S	17 I	25 S

### Comparison of colony morphology and clonality between wild-type and CHG-induced mutant strains

**Fig. 1:**
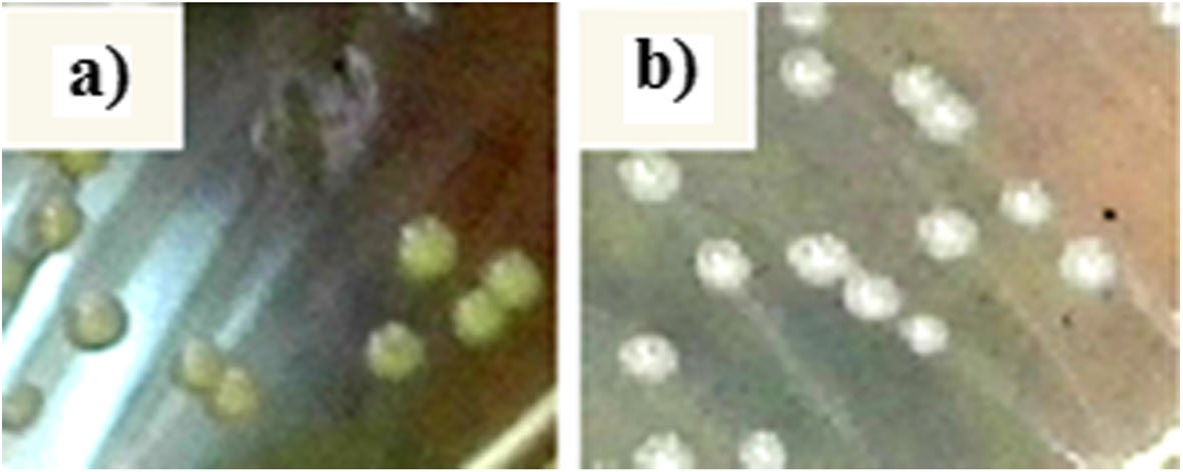
Changes in colony morphology from the pure cultures that occurred after CHG treatment on BHI agar plates. **a** Yellow colony in wild-type VAN-S strain; **b)** White colony in CHG-induced CHG_Van-I_ strain

The number and the location of the bands resulting from the restriction enzyme effect on the bacterial DNA were identical before and after serial exposure to CHG. Therefore, the same PFGE pattern verified that VAN-S and CHG_Van-I_ strains were clonal. However, colony pigmentation in the pure culture of *S. aureus* changed after CHG exposure on BHI agar plates. Wild-type VAN-S had yellow colonies (Fig. 1a), while VISA mutant CHG_Van-I_ had white colonies (Fig. 1b).

### CHG-induced gene expression changes

**Fig. 2:**
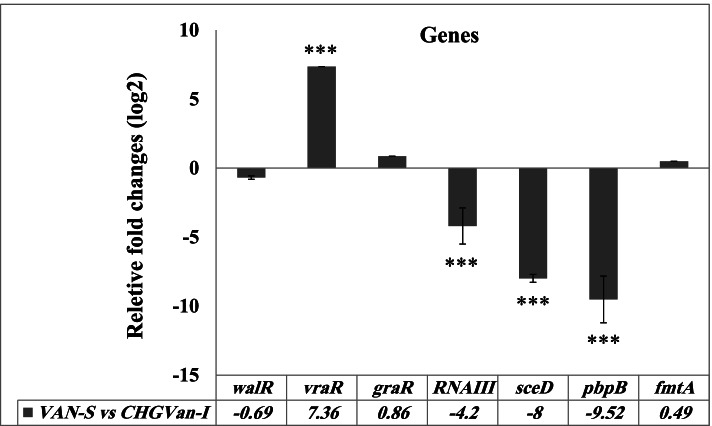
RT-qPCR analysis of the candidate genes involved in the VISA mechanism, including *gra*TSR, *vra*TSR, *wal*KR, *agr* RNAIII, *sce*D, *pbp*B, and, *fmt*A in VAN-S strain versus CHG_Van-I_. The results are presented as fold-change using REST2009 software. Error bars show standard deviation (± SD) from three independent trials. Significant differences (*P* ≤ 0.001) are shown with the *** symbol

RT-qPCR analysis revealed significant (*P* ≤ 0.001) *vra*TSR upregulation (+ 7.36-fold) and *pbp*B (- 9.52-fold), *sce*D (- eightfold), and *agr*RNAIII (- 4.2-fold) downregulation in CHG-induced mutant strain compared to wild-type VAN-S. There were no significant differences in the expression of *gra*TSR, *wal*KR, and *fmt*A between VAN-S and CHG_Van-I_ (see Fig. 2).

### Sequencing of *vra*T and *var*S genes involved in VISA mechanism

Complete sequencing of *vra*S and *var*T genes using the Sanger sequencing method was revealed six synonymous SNPs in *vra*S [T381C (encodes Asparagine), T456A (encodes Alanine), C870T (encodes Aspartic acid)] and *vra*T [T372C (encodes Arginine), T411C (encodes Isoleucine), G507A (encodes Valine)] genes in VAN-S compared to CHG_Van-I_. These mutations were located in the protein-coding DNA sequence (CDS) regions of genes. However, the new codons (in CHG_Van-I_) encoded the same amino acids (synonymous SNPs) with codons in the wild-type strain (VAN-S).

No non-synonymous substitution mutation was observed upon CHG serial exposure.

The gene sequences of *vra*S and *var*T genes in VAN-S and CHG_Van-I_ strains are available in the GenBank database (https://www.ncbi.nlm.nih.gov/) under the accession numbers MN508198, MN508199, MN503671, MN503672, respectively.

### Induced VISA-like features after CHG treatment

**Fig. 3:**
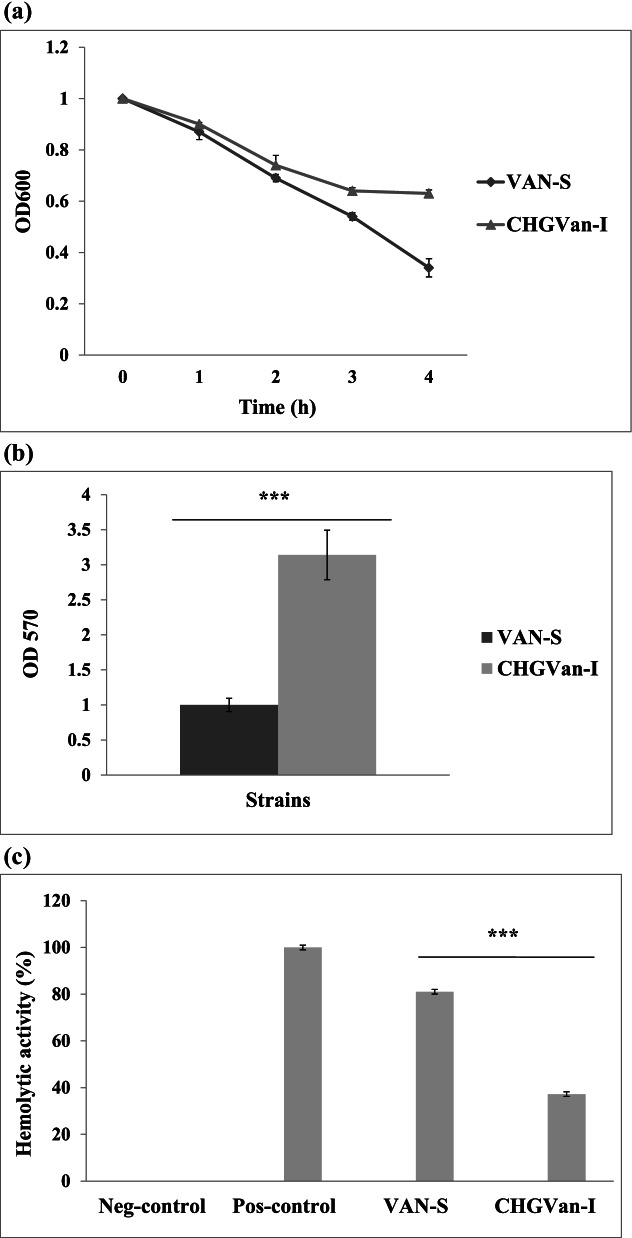
Kinetic and biofilm formation comparisons between wild-type (VAN-S) strain and CHG-induced (CHG_Van-I_) mutant. Error bars show standard deviation (± SD) from three independent trials. A *P* value ≤ 0.05 was considered a significant difference between VAN-S and CHG_Van-I_. The symbol *** shows significant differences with a *P* ≤ 0.001; **a)** Autolysis kinetic was analyzed by optical density measurement (at 600 nm) of bacteria cells (OD600 of 1) supplemented with 0.1% Triton X-100 at one-hour intervals. No significant differences (*P* ˃ 0.5) were observed in the autolysis rate using parametric paired-sample Student’s *t*-test; **b)** Biofilm formation of bacteria cells (107 CFU/m) supplemented with 0.5% (w/v) glucose were measured at OD570 by the 96-well plate method and 0.1% crystal violet staining. Biofilm formation raised 3.14-fold in CHG_Van-I_ using the paired-sample Student’s *t*-test; **c)** Alpha-hemolysin activity of the supernatant of healthy human RBCs and *S. aureus* (OD600 of 0.3) mixture (50:1) was measured at the OD543 using a two-tailed Wilcoxon signed-rank test analysis after one-hour incubation at 35 ± 2 °C. Diluted RBCs in PBS solution and Triton X-100 were used as the positive and negative controls. The hemolytic activities in VAN-S and CHG_Van-I_ were 81.03% and 37.2% (was reduced), respectively

Statistically, there was a significant (*P* ≤ 0.001) difference in the growth parameter, biofilm formation, and hemolytic activity between the wild-type strain and CHG-induced strain. However, no remarkable changes (*P* ˃ 0.5) were observed in the autolysis rate (Fig. 3a).

Doubling times in VAN-S was 27.28 min, while for CHG_Van-I_ was 39.23 min.

Biofilm formation increased 3.14-fold in CHG_Van-I_ (Fig. 3b).

The percentage of hemolytic activity in VAN-S was 81.03%, while in CHG_Van-I_ was reduced to 37.2% (Fig. 3c).

## Discussion

CHG bathing is a prevention strategy in patients with the central venous catheter to reduce hospital-acquired bloodstream infection with resistant organisms [19]. However, increased resistance to CHG and cross-resistance between CHG and antibiotics (such as ciprofloxacin) in clinical *S. aureus* isolates has been reported [3, 20, 21]. Recently, reduced susceptibility to VAN after *S. aureus* exposure to the sub-inhibitory concentration of CHG is taken into consideration [3]. In the present study, we observed that exposure to the supra-inhibitory CHG concentration could also induce VISA development in addition to reduced CHG susceptibility. Therefore, we confirmed that the emergence of VISA strains by cross-resistance mechanisms can be a particular concern with the improper use of CHG. In cross-resistance, resistance to more than one antimicrobial agent is developed by a similar mechanism of action [22].

The multidrug efflux pumps, mobile genetic elements, and changes in the cell membrane are the main mechanisms of CHG resistance [1, 2]. On the other hand, genetic mutation, particularly in transcriptional regulatory genes (*gra*SR, *vra*TSR, and *wal*KR), is the mechanism of VISA development [7]. Using RT-qPCR analysis, expression changes under CHG selection occurred in *vra*TSR and *pbp*B (a cell wall biosynthesis gene, which is regulated by the *vra*TSR system) genes were identified in this study. Therefore, we have assumed a role for *vra*TSR in CHG susceptibility in *S. aureus*. Although gene expression changes were detected in the mutant strain (CHG_Van-I_) compared to the wild-type, the identified mutations in *vra*S (T381C, T456A, C870T) and *vra*T (T372C, T411C, G507A) genes of CHG_Van-I_ were synonymous SNPs. The synonymous SNPs are a source of genome variability. Conventionally, these mutations do not affect the protein sequence because of the change of a codon to one that encodes the same amino acid. Although synonymous mutations might be expected to have no detectable effect on fitness or the protein function in which they occur, growing evidence suggests even changing the codon without amino acid change can cause alterations in phenotype, gene expression, and fitness. These changes can presumably occur through modifications to the rate or accuracy of transcription or translation, even changes to mRNA stability, protein folding, or protein secondary structure. Therefore, it suggests that synonymous mutations can be a cause of adaptive development and evolutionary dynamics [23, 24]. In the present study, allelic replacement experiments need to estimate the effect of the identified synonymous mutations (in *vraTSR*) on gene expression and phenotypic changes. In addition, it should be noted that other mutations (synonymous and/or non-synonymous) in different parts of the genome and changes in quorum sensing due to these mutations could be involved in the detected gene expression and phenotypic changes [7]. Therefore, whole-genome sequencing, total RNA sequencing, and future experiments need to investigate the cause of phenotypic adaptation and gene expression change.

In the recent study on CHG treatment of *S.aureus*, we also indicated altered expression and deleterious mutations in *S. aureus vra*TSR and *wal*KR [3]. However, it remains to be confirmed using allelic replacement experiments or site-directed mutagenesis. Surprisingly, the *vra*TSR expression was reduced in our previous study [3], but it was increased in the present study. The changes in CHG concentration or different genotypes of the wild-type might modulate the different *vra*TSR responses. However, *pbp*B expression was reduced in both studies [3]. It remains to be determined how *vra*TSR responds to CHG. The early studies determined the interaction of CHG with the bacterial cell membrane [25]. Since VraTSR regulates the cell wall biosynthesis pathway [26], our studies proposed the hypothesis of the effect of CHG on *S. aureus* cell walls. In *Enterococcus faecium*, Bhardwaj et al. (2016) suggested that CHG induces the expression of the *van*A resistance gene and other genes that alter cell wall synthesis, leading to a VRE/VanA phenotype [27].

We also identified *agr*RNAIII and *sce*D expression changes in the CHG-induced strain, compared to its wild-type strain. Downregulation of these genes has previously been associated with reduced autolysis and VISA development [7]. However, we did not observe significant changes in the autolytic activity of CHG-induced strain (CHG_Van-I_).

In the present study, other VISA phenotypic features were observed in CHG-induced *S. aureus* with reduced VAN susceptibility. CHG_Van-I_ had significantly lower growth rates than the wild-type strain, indicating that CHX adaptation could alter the ability of *S. aureus* to replicate and survive. To survive and escape from the immune system, VISA isolates reduce hemolytic activity and increase biofilm formation [5, 28]. Here, a significant decrease in hemolytic activity occurred in CHG_Van-I_ compared to wild-type VAN-S, and biofilm formation was increased after serial CHG exposure. There are also reports of the induced biofilm formation of bacteria in the presence of CHG [29, 30], although CHG is used to prevent biofilm formation in medical applications [31]. Therefore, improper CHG concentrations could lead to increased biofilm formation, as previously was proposed. Extracellular DNA (with a negative charge) that presents in the biofilm can bind to CHG (a cationic agent) [32]. This mechanism can induce increased resistance to antimicrobial agents than the planktonic phase [33].

A VraS⋅N340-D347del novel mutation was previously reported in a VISA drove of sub-MIC CHG treatment [3]. Here, despite phenotypic and gene expression changes in exposure to an inappropriate concentration of CHG, any significant mutation was not observed in suspected sequenced genes.

The main limitation of the present work is that we did not perform control genetic experiments using whole-genome sequencing and total RNA sequencing. Moreover, the deletion studies of *vra*TSR and *pbp*B genes after exposure to CHG were not performed to assess their contribution to CHG susceptibility. Further studies in these areas are proposed.

## Conclusions

The present study has implications in health care settings. The improper concentration of CHG (supra- or sub-inhibitory) may be a risk factor for VISA development in the clinic, contributing to VAN treatment failures on *S. aureus* in these cases. Therefore, CHG should be under health supervision where CHG bathing is utilized in hospital wards. The combination therapy with CHG may avoid the selection of VISA isolates. Moreover, the induction of VISA by CHG confirmed the possible role of CHG in cell wall biosynthesis pathways, including may through the vraTSR locus. These subjects proposed will be a focus of future study.

## Data Availability

The sequences of *vra*S and *vra*T genes in VAN-S and CHG_Van-I_ strains have been recorded in the NCBI GenBank database (https://www.ncbi.nlm.nih.gov/) with accession numbers MN508198, MN508199, MN503671, and MN503672.

## Abbreviations

CHG: Chlorhexidine gluconate
*S. aureus*: *Staphylococcus aureus*
VISA: Vancomycin-intermediate *S. aureus*
VAN: Vancomycin
MIC: Minimal inhibitory concentration
CHG_Van-I_ strain: CHG-induced mutant
VAN-S: Wild-type strain
PFGE: Pulsed-field gel electrophoresis
RT-qPCR: Reverse Transcription quantitative PCR
OD_660_: Optical density at 660 nm
PBS: Phosphate-buffered saline
RBC: Human red blood cell
